# Treatment and outcomes in patients with left ventricular thrombus - experiences from the Aga Khan University Hospital, Nairobi - Kenya

**DOI:** 10.11604/pamj.2021.39.212.28585

**Published:** 2021-07-23

**Authors:** Mohamed Hasham Varwani, Jasmit Shah, Mzee Ngunga, Mohamed Jeilan

**Affiliations:** 1 Department of Internal Medicine, Aga Khan University, Nairobi, Kenya

**Keywords:** left ventricular thrombus, cardioembolic, directly acting oral, anticoagulants, left ventricular, systolic dysfunction, stroke

## Abstract

**Introduction:**

left ventricular thrombus (LVT) may lead to thromboembolism and has been associated with increased morbidity and mortality. Little is known about the incidence, etiology and outcomes in patients with LVT in Africa. The objective was to determine the etiology, treatment practices, rate of resolution and clinical outcomes in patients with LVT in the region.

**Methods:**

a review of all echocardiograms performed in 2017 and 2018 at the Aga Khan University Hospital, Nairobi was carried out and patients with LVT identified. Physician review of charts was performed to document clinical characteristics and outcomes.

**Results:**

during the study period 100 patients with LVT were identified (1.3% of adult echoes). The mean LVEF was 28.5% (±11.0%) and 88 (88%) patients had an LVEF of less than 40%. Underlying etiology of LV dysfunction was post myocardial infarction (MI) in 28 (28%), chronic ischemic cardiomyopathy in 42(42%) and non-ischemic cardiomyopathy in 30 (30%) patients. In 15 (15%) patients a stroke or TIA predated the diagnosis of LVT. Long term anticoagulation was given to 92 (92%) patients. Among these, 34 (37%) received warfarin while 58 (63%) were treated with a DOAC. In the 64 patients who had reassessment imaging (median duration 177 days), complete thrombus resolution was noted in 38 (59.4%). One-year clinical outcome data was available for 85 patients: 13 (15.3%) patients had died, 4 (4.7%) had suffered a stroke, and 8(9.4%) had had a bleeding episode. Rates of thrombus resolution (warfarin 64%, DOAC 55.6%, p=0.51), stroke (warfarin 2.9%, DOAC 1.7%, p=1.0) and bleeding (warfarin 5.9%, DOAC 5.2%, p = 1.00 were not significantly different among patients treated with warfarin and DOAC.

**Conclusion:**

we noted a high incidence of LVT compared to contemporary Western series. The majority of our patients were treated with DOACs. There were no significant differences in outcomes between patients treated with a DOAC and those receiving warfarin. Prospective evaluation on the efficacy and safety of DOACs for this indication is needed.

## Introduction

Development of left ventricular thrombus (LVT) in patients with systolic dysfunction is associated with systemic embolism and increased morbidity and mortality [1, 2]. Most series have studied LVT following myocardial infarction. The incidence of LVT in developed countries has been on a downward trend [3]. In the 1980s, the reported incidence of post MI LVT was 17% overall, and up to 46% in patients with anterior MI. A recent meta-analysis of more than 10,000 patients examining the incidence of LVT in the era of primary percutaneous coronary intervention (PCI) found the incidence to be 3% overall and 9% in anterior MIs [4]. LVT in the setting of non-ischemic cardiomyopathy has been much less studied. The incidence ranges between 11-44%. Reported clinical outcomes in patients with LVT vary. A 1988 study of serial echoes in 60 patients with LVT post MI reported a thrombus resolution rate of 40% during a mean follow-up of 24 months. Embolization occurred in 14% and death in 31% [5]. In a more recent retrospective study of 128 patients thrombus resolution occurred in between 40% to 100% of patients on repeat imaging at one year depending on the agent used for anticoagulation, embolic events in only 1.9% and death in 13% [6]. Traditionally warfarin has been used for anticoagulation in patients with LVT. Recently, several case series and observational studies have reported on the successful use of DOACs for this purpose [7, 8]. DOACs provide an attractive alternative to warfarin given the lack of need for therapeutic monitoring but more data is needed to establish both efficacy and safety. The 2017 ESC guidelines for STEMI recommend treatment of LVT with oral anticoagulation for up to six months guided by repeated imaging, but no agent preference is given [9].

Little is known about the incidence and etiology of LVT in sub-Saharan Africa. Cardiovascular epidemiology on the continent is experiencing a transition and is uniquely affected by challenges in availability of expertise and underdeveloped systems of care [10, 11]. There is a growing prevalence of ischemic heart disease and acute coronary syndromes [12]. Unfortunately, delayed presentation and missed MI is a common problem, and primary PCI remains unavailable to the vast majority of the population [13]. This study has been designed to document the echocardiographic and clinical outcomes of LVT in a sub Saharan African population, to gain insight into the underlying risk factors, study local treatment practices, and to evaluate long term outcomes.

## Methods

### Study design and setting

This was a two-year retrospective observational study carried out at the Aga Khan University Hospital Nairobi, Kenya.

### Study population

All adult patients (18 years and above) who had an echocardiogram diagnosis of LVT in the years 2017 and 2018 were included in the study.

### Data collection

Echo reports for 2017 and 2018 performed at the Aga Khan University Hospital, Nairobi were reviewed to identify patients with LV thrombus. Patient charts, electronic medical records and the Heart Clinic registry was used to collect clinical and outcome data. Both admitted patients and ambulatory patients referred for echocardiogram were included.

### Study procedures

Echocardiograms had been performed on GE Vivid 7 and GE Vivid Q echo machines. Echo data was analyzed on GE EchoPac software and reported by consultant cardiologists with at least level II echo accreditation. The ejection fraction was calculated using Simpson´s Biplane method. Wall motion score (WMS) was calculated using the American Society of Echocardiography 16 segment model and segmental scores assigned as follows: normal - 1, hypokinesia - 2, akinesia - 3, dyskinesia or aneurysmal - 4. The WMS was calculated as the sum of the scores for the 16 segments. The wall motion score index (WMSI) was calculated by dividing the WMS into 16. The underlying etiology predisposing to LVT formation was classified as post-MI if the LVT diagnosis was made within 3 months of a coronary event. If the MI occurred more than three months prior to the diagnosis of LVT, or if coronary artery disease was deemed to be the underlying cause of LV dysfunction then this was classified as chronic ischemic cardiomyopathy. LV dysfunction in the absence of coronary artery disease was categorized as non-ischemic cardiomyopathy. LV thrombi were classified as either layered (concave) or cavitary (convex) depending on the relationship of the cavitary surface of the thrombus to the underlying endocardium. The size of LVT was measured in the maximum dimension in the echo view that showed the largest portion of the thrombus. Layered thrombi dimensions were measured along the cavitary surface. Thrombus resolution was defined as complete disappearance of a previously noted LV thrombus on a follow-up echo study. Bleeding was categorized according to the Bleeding Academic Research Consortium (BARC) standardized definitions.

### Data analysis

IBM SPSS version 23 was used for data analysis. Continuous variables are expressed as mean ± standard deviation. Categorical variables are expressed as percentages. For between group comparisons Chi square and Fisher´s exact test has been used for categorical variables, and the Student´s t-test and ANOVA for continuous variables. Comparison of medians was performed using the Mann-Whitney U test.

### Ethical considerations

The study complies with the declaration of Helsinki and received approval from the Aga Khan University Institutional Ethics Review Committee.

## Results

Between January 1^st^ 2017 and December 31^st^ 2018, a total of 7561 adult transthoracic echocardiograms were performed in our echo lab for a variety of indications. Of these, 100 (1.3%) studies revealed the presence of an LVT. The underlying cause of LV dysfunction in these patients was Post MI in 28% of the patients, chronic Ischemic cardiomyopathy in 42% and non-ischemic cardiomyopathy in 30%. For the baseline characteristics a full review of medical records was available for all patients with LVT.

### Demographic and clinical characteristics

Table 1 summarizes the demographic and clinical characteristics of the patient groups. 77% were male, and mean age at diagnosis was 60.9 years (SD 14.1). Forty-seven percent (47%) were diabetic, 61% hypertensive and 21% were current smokers. Twenty-percent percent (23%) and 4% had a history of remote PCI and CABG respectively. Of the 70 patients with LVT related to coronary artery disease, 28 had suffered a myocardial infarction in the last three months, whereas 42 had a history of a more remote MI or an evaluation in keeping with ischemic cardiomyopathy. Among patients with post MI LV thrombus 21 (75%) had presented with a STEMI and 7 (25%) with non-ST-elevation acute coronary syndromes (NSTE-ACS). During the period of the study the number of admissions with STEMI and non-STEMI (NSTEMI) to the hospital were 136 and 162 respectively. This translated to an incidence of post MI LVT of 15.4% for STEMI and 4.3% for NSTEACS. The median time to presentation from symptom onset was 12 hours for STEMI and 24 hours for NSTEMI. Delayed presentation was common and 11 of the 28 patients (39.3%) presented more than 12 hours after symptom onset. The median duration from onset of symptoms to echo diagnosis of LVT in patients with AMI was 3 days (range 1 - 48 days, IQR 4 days). In patients with STEMI, 19 (90.5%) had anterior ST segment elevations while 2 (9.5%) had inferior STEMI. The mode of reperfusion in 16 (76.2%) of the patients was primary PCI and 4 (19%) received thrombolysis. One patient did not receive any reperfusion therapy due to late presentation and resolved chest pain on arrival. All 21 patients with STEMI had coronary angiography. Left anterior descending artery (LAD) was the culprit vessel in 17 patients (81%), ramus intermedius in one patient (4.7%), and the RCA in 3 patients (14.3%).

**Table 1:** clinical characteristics of patients with left ventricular thrombus

	Post MI (n = 28)	Chronic ischemic CMP (n = 42)	Non ischemic (n = 30)	P value
Age	61.2 (SD 13.2)	65.4 (SD10.8)	54.2 (SD 16.5)	0.03
Males (%)	78.6	85.7	63.3	0.08
BMI	26.5 (3.4)	29.0 (5.6)	26.8 (4.9)	0.06
Diabetes (%)	46.4	61.9	26.7	0.01
Hypertension (%)	46.4	78.6	50	0.01
Current Smokers (%)	23.8	26.2	16.7	0.13
Creatinine Clearance (ml/min/1.73m3)	74.4	71.0	78.7	0.60
Prior (remote) ACS (%)	21.4	64.3	n/a	<0.001
Prior (remote) PCI (%)	14.3	73.8	n/a	<0.001
Prior CABG (%)	3.6	7.1	n/a	0.31
Prior VTE	0	0	0	1.0

MI - myocardial infarction, CMP - cardiomyopathy, BMI - body mass index, ACS - acute coronary syndrome, CABG - coronary artery bypass grafting, VTE - venous thromboembolism.

### Echo characteristics

The mean LVEF using Simpson´s Biplane method was 28.5% (SD 11.0%) and 88 (88%) patients had an LVEF of < 40%. The mean LVEF in patients with non-ischemic cardiomyopathy (23% SD 9.4) was significantly lower than in patients who were post-MI (31.8% SD 10.2) and with chronic ischemic cardiomyopathy (30.2% SD 11.2). The mean LV end diastolic and end systolic diameters were 54.7mm (SD9.4) and 44.9mm (SD11.9) respectively. The mean wall motion score index (WMSI) was 2.1(SD 0.4). A single thrombus identified in 97 patients, while 3 patients had two thrombi each. The size of the thrombi ranged from 6mm to 43mm, with a mean of 19.7 (SD 6.4) mm. 97 (94.2%) of the thrombi were located in the apex, 4 (3.9%) along the inferior wall and 2 (1.9%) along the septum. 26 (25.2%) of the LVT were classified as layered and 77 (74.8%) were cavitary.

### Treatment practices

Of the 100 patients diagnosed with an LVT, 92 received long term anticoagulation therapy. Two patients did not receive long term anticoagulation due to presence of bleeding ulcers in both, and 6 patients died prior to discharge from hospital and were treated with enoxaparin but not transitioned to oral therapy. Of the patients treated with long term anticoagulation, 34 (37%) were treated with warfarin while 58 (63%) received a directly acting oral anticoagulant (DOAC). Among the DOACs, rivaroxaban was used in 46 (79.3%), dabigatran in 7 (12.1%) and apixaban in 5 (8.6%) patients. DOACs were the most frequently used treatment across all patients´ groups: 50% among post MI and non-ischemic CMP, and 69% among patient with chronic ischemic CMP. During the one year of follow-up, 84 INR readings were available for the patients who were on warfarin. Only 11 of these readings were between 2.0 - 3.0 (time in therapeutic range 13.1%). This was however limited to International Normalized Ratio (INR) monitoring that was done within the university lab. INR tests performed at external labs were not available for this analysis.

### Echocardiographic outcomes

**Figure 1:**
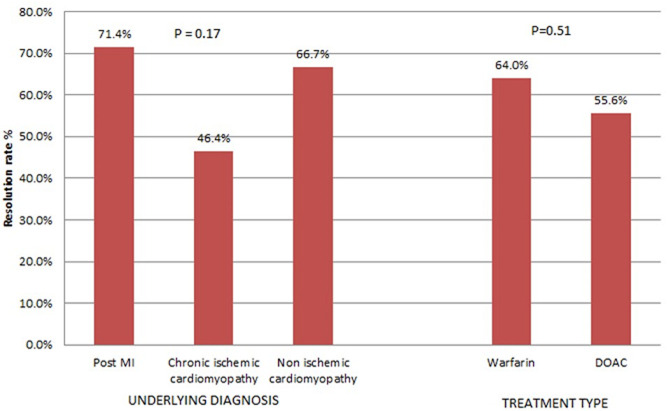
rate of thrombus resolution by underlying diagnosis and treatment type

A reassessment Echo was done in 64 (64%) patients at a median duration of 177 days after the initial echo. Complete LVT resolution occurred in 38 (59.4%) of the patients and persisted in 26 (40.6%). In those with LVT persistence a reduction in thrombus size was noted in 19 (29.7%) of the patients and an increase in 7 (10.9%). There was no statistically significant difference in thrombus resolution rates by underlying diagnosis and treatment type. This is demonstrated in Figure 1. On reassessment 50 (78.1%) patients had an improvement in LVEF (mean increase of 8.3%), 5 (7.8%) had no change in EF, and 9 (14.1%) had a reduction in EF (mean reduction of -7.7%). In patients who had thrombus resolution, the mean EF improved significantly more than in those with thrombus persistence (7.3% vs 2.6% n=0.016).

### Clinical outcomes

At one-year clinical outcome data was available for 85 patients. 13 (15.3%) patients died during the first year after diagnosis of LVT. The median duration from diagnosis of LVT to death was 16 days. In 15 (15%) patients the diagnosis of stroke or TIA predated the diagnosis of LVT, which was discovered during echo for evaluation of the stroke. Among these patients 8 (53.3%) had underlying non-ischemic CMP, 6 (40%) had chronic ischemic CMP, and one patient (6.7%) had a recent MI. Following an echo diagnosis of LVT, only four patients (4.7%) developed thromboembolism (stroke in all 4) during the first year. These events occurred on days 1, 2, 4 and 38 respectively. Table 2 summarizes characteristics of these patients. Documented clinically significant bleeding occurred in 8 patients in the first year. The site of bleeding was intracranial in 4 patients, gastrointestinal in 3 and genitourinary in 1 patient. Bleeding severity was classified as BARC2 in 3 patients, BARC 3C in 2, and BARC5 in 3. The median duration to bleeding was 90.5 days. At the time of developing a bleed, 2 patients were on warfarin, 3 on DOAC, 2 on enoxaparin and 1 patient was not on any treatment. This patient had a pre-existing history of bleeding ulcers. There was no significant difference between the proportion of patients on warfarin and DOAC who developed bleeding (5.9% and 5.2% respectively p = 0.85).

**Table 2:** characteristics of the patients who developed stroke in the first year after diagnosis of left ventricular thrombus

	Patient 1	Patient 2	Patient 3	Patient 4
**Age**	28	61	24	88
Gender	Male	Male	Male	Male
Underlying diagnosis	Post MI (STEMI)	Post MI (STEMI)	Non ischemic CMP	Post MI (STEMI)
LVEF	38	30	10	20
Maximum dimension of thrombus(mm)	23	17	25	12
Heart rhythm on ECG at time of stroke	Sinus rhythm	Sinus rhythm	Sinus rhythm	Sinus rhythm
Type of thrombus	Cavitary	Cavitary	Cavitary	Layered
Treatment on discharge	Rivaroxaban	Rivaroxaban	Warfarin	Rivaroxaban
Treatment during stroke	Enoxaparin	Enoxaparin	Warfarin	Rivaroxaban
Days after diagnosis	2	1	38	4
Alive at one year	yes	yes	yes	Lost to follow-up (32 days)

MI - myocardial infarction, STEMI - ST segment elevation myocardial infarction, CMP - cardiomyopathy, ECG - electrocardiogram, LVEF - left ventricular ejection fraction.

Of the 92 patients discharged on long term therapy follow-up data on treatment duration was available for 78 patients. Treatment duration varied from 3 months to more than a year. It was noted that the majority of the patients (60.3%) received long term treatment which lasted more than a year. Patients with LVT persistence on echo were more likely to get treatment beyond one year (80%) compared to those who had no reassessment echo (61.1%) and those who had LVT resolution (45.7%). Therapy was switched from warfarin to DOAC in 4 patients at a median duration of 185 days. The reason for this change was documented as labile INR in two patients, and patient convenience for the other two. No patients were changed from DOAC to warfarin during the one year of follow-up.

### Comparison of warfarin and DOAC

**Figure 2:**
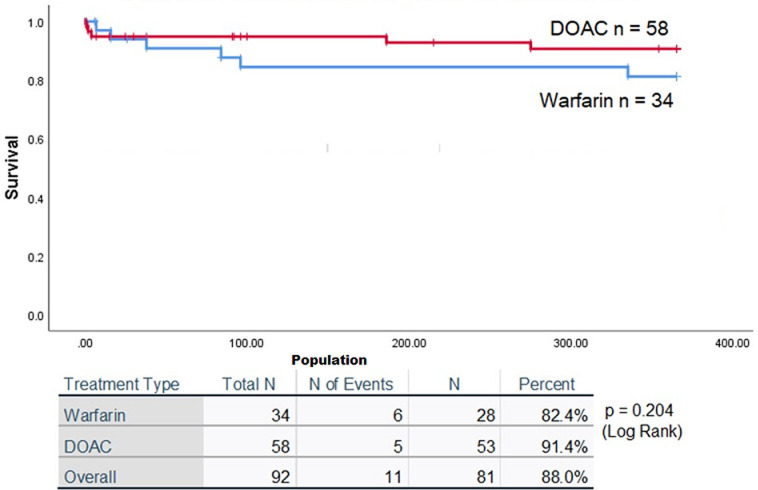
comparison of outcomes in patients treated with warfarin and directly acting oral anticoagulants

Among patients who received long term anticoagulation with warfarin and DOAC, the rate of thrombus resolution on reassessment echo, and stroke and bleeding at one year are shown in Table 3. The difference in the outcomes was not statistically significant. Survival analysis was performed using Kaplan Meier estimates. At the end of one year, stroke and systemic embolism free survival was 82.4% and 91.4% for warfarin and DOAC respectively (p=0.204). This is shown in Figure 2.

**Table 3:** comparison of outcomes in patients treated with warfarin and directly acting oral anticoagulant (DOAC)

Echo Outcomes	Warfarin (n=25)	DOAC (n=36)	p
Thrombus resolution	16 (64.0%)	36(55.6%)	0.51
(Median duration to echo)	178 days	149 days	0.95 Mann-Whitney U
Clinical outcomes	Warfarin (n = 34)	DOAC (n = 58)	
Stroke	1 (2.9%)	1 (1.7%)	1.0
Bleeding	2 (5.9%)	3 (5.2%)	1.0

DOAC - directly acting oral anticoagulant

## Discussion

To our knowledge this is the first African study that reports on long term clinical and echocardiographic outcomes in patients with LVT. An incidence of 1.3% in this two-year review of 7561 adult echo studies is much higher than contemporary series which report incidence of 0.09% - 0.18% in their echo databases [3, 12]. The indications for echo, proportion of normal echo studies and clinical profiles of these populations are probably varied and may explain this difference partially. However, delayed presentation of acute coronary syndromes and subsequent development of heart failure could also contribute to this higher incidence. In contrast to these studies from Europe and North-America, two Nigerian series published in 2014 and 2016 revealed a much higher incidence of 8.9% and 7.8% respectively [13, 14]. The high prevalence of peripartum cardiomyopathy in Nigeria could partially explain this disparity in incidence of LVT. The findings of these series are summarized in Table 4 [3, 12-14].

**Table 4:** a non-comprehensive summary of African and contemporary North American and European left ventricular thrombus series

Authors, Country, Year	Type of study	Incidence	Main underlying etiologies	Treatment	Long term outcomes
McCarthy *et al*. USA, 2019 [3]	Retrospective, Echo and chart review	128 of 140,636 echoes reviewed (0.09%)	Heart failure 68.5% AMI 25.9%	Warfarin 87%, LMWH 9.3%, DOAC 3.7%	Thrombus resolution in 75% of warfarin group, 40% LMWH, 100% in DOAC group
Lattuca *et al*. France, 2020 [12]	Retrospective, Echo and chart review	159 of 90,065 echoes reviewed (0.18%)	CAD 78.6% (Post MI 46.5%) DCM 14.5%	VKA 48.4%, Heparins 27.7%, DOAC 22.6%	LVT resolution 62.3% (median 103 days), mortality 18.9%, stroke 13.3%, major bleeding 13.2% (median follow-up 632 days)
Talle *et al*. Nigeria, 2014 [13]	Retrospective, Echo and chart review	84 of the 949 abnormal adult echoes (8.9%)	DCM 39.3% Post MI 29.8% PPCM 21.4%	Warfarin, UFH and LMW (proportions not defined)	Not defined. 13.1% presented with thromboembolic complications
Saidu *et al*. Nigeria, 2016 [14]	Retrospective, Echo and chart review	79 of 1012 echoes (7.8%)	PPCM 77.2% DCM 12.7%	Not defined	Not defined. 53.2% presented with thromboembolic complications
Current study - Varwani *et al*. Kenya, 2020	Retrospective, Echo and chart review	100 of 7561 echoes reviewed (1.3%)	Post MI in 28%, Chronic Ischemic cardiomyopathy 42%, non-ischemic cardiomyopathy30%.	Warfarin 37%, DOAC 63%	LVT resolution 59.4% (median 177days), mortality 13%, Stroke 4.7&, Bleeding 9.4%

DCM - dilated cardiomyopathy, MI - myocardial infarction, PPCM - peripartum cardiomyopathy, UFH - unfractionated heparin, LMWH - low molecular weight heparin, DOAC - directly acting oral anticoagulant, CAD - coronary artery disease, LVT - left ventricular thrombus.

The underlying etiology for LV dysfunction was coronary artery disease in the majority of the patients, with 28% having been treated for an acute coronary syndrome in the three months preceding the diagnosis, and 42% having had a remote coronary event or LV dysfunction attributed to coronary artery disease. This is comparable to other contemporary series but contrasts with previously published African data in which non-ischemic dilated cardiomyopathy represents a much larger proportion of the patients. It should be borne in mind that many patients with dilated cardiomyopathy in Kenya are managed medically by physicians in peripheral hospitals, while patients who have an acute coronary syndrome are more likely to be referred to cardiac centers for evaluation and treatment. This may partially explain the higher proportion of patients with CAD in our series. Delayed presentation in patients with acute coronary syndromes was common - the time from symptom onset to hospital arrival was more than 12 hours in nearly 40% of the patients. This is a common challenge in Kenya and Africa. Lack of recognition of symptoms by patients and caregivers, poor referral networks and the presence of specialized centers exclusively in urban centers are some of the contributing factors [11].

The potential for developing stroke and other thromboembolic phenomena is the main concern in patients with an LVT. In our series LVT was discovered in 15% during evaluation of stroke. The majority of these patients had an underlying non ischemic dilated cardiomyopathy. The role of serial cardiac imaging in patients with chronic cardiomyopathies for the detection of LVT has not been defined. Following a diagnosis of LVT and institution of anticoagulation therapy only 4 patients developed a stroke. 3 of these events occurred early (between 1 - 4 days) of diagnosis of LVT in the post MI period. One event occurred in a patient with non-ischemic cardiomyopathy on day 38. Other than stroke, no other embolic phenomena were documented.

Complete resolution of LVT occurred in 59.4% of the patients who had a reassessment echocardiogram at a median duration of 177 days. Similar rates have been documented in other series. It is unclear why LV thrombus persists in some patients despite anticoagulation. Suboptimal anticoagulant dosing, non-compliance and persistence of predisposing factors such as low LVEF, LV aneurysm and regional akinesis could be contributing factors. On the other hand, thrombi may become organized and covered by an endothelial lining and appear to persist on reimaging. Theoretically the embolic potential of such thrombi would be low. There is no consensus on the duration of anticoagulation in patients with LVT. Guidelines suggest 3 to 6 months of anticoagulation guided by re-imaging, but the evidence base for this is wanting [8, 15]. In this series, more than 60% of the patients received treatment beyond one year. Fear of thromboembolism and the devastating consequences of a potential stroke may sway clinicians and patients towards long term, sometimes even lifelong anticoagulation. The embolic potential of persistent LVT beyond six months of treatment has been poorly studied, and it is unknown whether the benefit of prolonged anticoagulation outweighs the risk of bleeding. In addition, predictors of recurrent LVT in patients with persistently depressed LVEF and large akinetic areas have not been well defined. Thus a decision to continue anticoagulation after LVT resolution should be guided by patient factors and serial imaging.

Among patients who received long term anticoagulation nearly two thirds (63%) were treated with DOACs. To date, this is one of the largest series of DOAC use for treatment of LVT. Case reports and observational series have suggested that these agents may be as effective as vitamin K antagonists in the treatment of LVT [6, 16-19]. In our series we found no significant differences in rate of thrombus resolution, stroke and bleeding among patients treated with warfarin and DOACs. This series furthers our knowledge on the use of DOACS for this indication. In post MI patients who have an indication for DAPT and anticoagulation, a broader evidence base exists for DOAC use and early de-escalation from triple therapy to DOAC and single antiplatelet [20].

This study represents one of the largest reports of LVT outcomes from Africa and provides novel observational data on the use of DOACs for treatment of LVT. However, this is a single center experience from a private teaching and referral hospital, and the findings may not be generalizable to the rest of the African population. It is well recognized that the sensitivity and specificity of echo in the diagnosis of LVT is inferior to other modalities such as contrast echo and cardiac MRI and this is one of the inherent weaknesses of this study.

## Conclusion

This study provides important insights into patterns of formation and resolution of LVT and clinical outcomes in patients in an African referral hospital. There is a much higher incidence of LVT compared to other contemporary series, and a similar rate of thrombus resolution. Most patients were treated with DOACs with similar outcomes to those treated with warfarin. DOACs may represent a convenient and viable therapeutic option for patients with LVT and should be subjected to controlled trials.

### What is known about this topic

- Left ventricular thrombus (LVT) is associated with morbidity and mortality in patients with left ventricular systolic dysfunction;
- Vitamin K antagonists have traditionally been used in the treatment of LVT;

### What this study adds

- This study report of a much higher incidence of LVT in the African context;
- Our study adds to the growing body of literature which suggests safe and effective utility of directly acting oral anticoagulants (DOACs) in the treatment of LVT;
